# The phase stability of InP(001) surfaces upon oxygen exposure from first principles

**DOI:** 10.1039/d5ra00855g

**Published:** 2025-03-18

**Authors:** Vibhav Yadav, Holger Euchner, Matthias M. May

**Affiliations:** a Universität Tübingen, Institute of Physical and Theoretical Chemistry D-72076 Tübingen Germany matthias.may@uni-tuebingen.de; b Universität Tübingen, Center for Light-Matter Interaction, Sensors and Analytics LISA+ D-72076 Tübingen Germany

## Abstract

III–V semiconductors such as indium phosphide and multinary alloys derived thereof have shown high performance in multi-junction photoelectrochemical devices for solar water splitting. However, electrochemical conditions, especially in aqueous electrolytes, often lead to changes in surface structure and stoichiometry. These changes then affect the electronic structure, for instance leading to the formation of charge-carrier recombination centers or points of attack for dissolution of the material. It is therefore important to understand the surface structures that may arise in electrochemical environments to identify routes for electronic and electrochemical surface passivation. In this work, we assess the impact of oxygen adsorption on surface reconstructions of InP(001) *via* first principle calculations. We observe predominantly P-rich surfaces for a large range of indium and oxygen chemical potentials, showing P_*x*_O_*y*_-type polyphosphate motifs. On the other hand, the frequently assumed In-rich (2 × 4) mixed-dimer surface reconstruction is found to be unstable for a large range of oxygen chemical potentials.

## Abstract 2

This first-principles study provides insights into the stability of oxygenated InP(001) surfaces.

## Graphical abstract

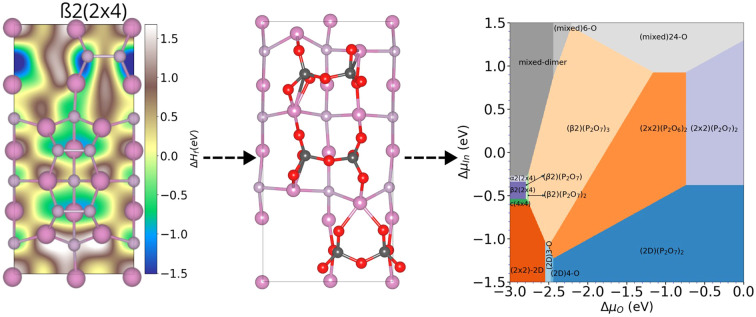

## 1 Introduction

Transitioning towards renewable energy sources to reduce the reliance on fossil fuels is crucial for limiting global warming.^1^ Hydrogen produced from solar water splitting, as a carbon-neutral fuel has had a substantial amount of research put-forth in the recent years.^2–4^ In particular, photoelectrochemical (PEC) water splitting is a promising option for hydrogen production. However, maintaining high solar-to-hydrogen efficiency over the necessary lifetime of the device remains a problem due to the semiconductor–electrolyte interface on the top photoabsorber.^5,6^ Hence, it is crucial to understand the underlying facets necessary to improve the sustenance of photoelectrodes for direct solar water splitting, where the photabsorber is in contact with the electrolyte.^4,7^

Limiting (photo)corrosion that can lead to mid-gap states resulting in charge-recombination is therefore essential for viable PEC devices. Here, suitable surface passivation can suppress surface states, reduce recombination, and protect the surface from corrosion, thus solving problems related to the device's instability.^4,8^ The first step for the knowledge-driven design of such a surface passivation is to understand the change in the electronic structure that may arise at the semiconductor surface under operating conditions. Here, Indium Phosphide (InP) is often applied as model system to gain insights into (photo)-electrochemical processes. InP is a (cubic) zinc-blende structured III–V semiconductor compound with electrical and optical properties that render it valuable for a range of opto-electronic applications, including photonic crystals,^9^ energy harvesting and storage.^10^ InP possesses a direct bandgap of 1.34 eV alongside high electron mobility. The material can be prepared epitaxially in high-quality, but also serves as a wafer-based substrate for a variety of opto-electronic devices.^11^ These properties make InP particularly attractive for high-efficiency solar cells and photoelectrochemical water splitting applications. For instance, when alloyed with gallium to form GaInP_2_, InP demonstrates promising solar-to-hydrogen conversion efficiencies as part of the absorber stack in tandem configurations, but also the charge-selective window layer in the form of Al_*x*_In_1−*x*_P.^4^ Due to the challenges that arise for the study of electrochemical systems under realistic conditions, both experimentally and computationally,^12,13^ a widely used approach is to study the surface chemistry in vacuum conditions under the supply of oxygen or water.^14–18^ These studies found the oxidation of InP to be a highly structure-sensitive process that exhibits distinct behaviour for In- and P-rich surfaces. The supply of molecular oxygen leads to oxygen insertion into In–In and In–P back bonds on the respective surfaces,^14^ while water preferentially interacts with surface In–P bonds.^15^ An in-depth computational study of Wood *et al.*^19^ was mainly based on the In-rich, mixed-dimer reconstruction of InP(001) and used adatom adsorption and density-functional theory (DFT)-based molecular dynamics calculations of the solid–liquid-interface. They found the predominant formation of In–O–In and In–O–P bonds for the mixed-dimer reconstruction, identifying In–O–In as being more detrimental due to in-gap electronic states.^19,20^ An adsorbate-assisted kinetic H_2_O dissociation was also suggested, together with a long-range Grotthuss mechanism of surface hydrogen migration, which could enhance proton adsorption and hydrogen evolution at different surface sites. Work on the ternary Al_*x*_In_1−*x*_P suggested symmetric oxygen distribution on the InP mixed-dimer reconstructions after adsorption or substitutive insertion.^16^ More complex motifs were found in a study combining near-ambient pressure photoelectron spectroscopy and DFT,^21^ but the exact starting surface of the experimental study was not well-established. The sputtering routine described and the low-energy electron diffraction patterns suggest that the starting point was a surface similar to the mixed-dimer surface with a relatively high density of defects.^17^ Surface defects, however, can qualitatively change the interaction of the III–V(001) surfaces with adsorbed water.^22^ A recent computational study also suggested that the interaction of InP surfaces with hydrogen is strongly influenced by substrate doping, and the surface hydrogen content will impact electronic properties such as Fermi level pinning.^23^

Surface chemistry studies of clean InP(001) reconstructions apart from the mixed-dimer reconstruction are, however, rather limited, both computationally and experimentally. While well-ordered interfaces between InP and aqueous electrolytes have indeed been demonstrated to exist, their exact nature is not yet established.^24^ In-rich and Cl-rich surfaces might be present in limited potential ranges. While our recent work using the computational hydrogen electrode suggests that H–Cl co-adsorption in these conditions may be thermodynamically limited under these conditions,^25^ we did not consider oxygen as ingredient for the surface phases. To understand possible surface oxidation pathways, it is therefore necessary to systematically assess the phase diagram of InP(001) with respect to oxygen chemisorption with a broad structural basis.

In this work, we therefore first analyse the stability of clean InP(001) stable surface reconstructions with respect to the surface constituents chemical potential *via* density functional theory. Next, for stable surface reconstructions, we study the resulting phase stability due to dissociative chemisorption of O_2_*via* two different approaches. We find that the phase stability of polyphosphate moieties along with the insertion of O-atoms into the underlying In–P back bonds at higher coverages is evident in the overall phase diagram.

## 2 Methods

### 2.1 Computational details

For structural relaxation, we employed the Gaussian and Plane Wave (GPW) method with DZVP basis set as implemented in the DFT code CP2K^26^ in combination with the non-local and norm-conserving GTH-PBE pseudopotentials. Exchange and correlation were accounted for *via* the generalised gradient approximation in the form introduced by Perdew, Burke, and Ernzerhof.^27,28^ Furthermore, the Grimme-D3 correction^29^ was added to consider van der Waals interactions in a pair-wise dispersion correction. After performing convergence tests to determine suitable layer and vacuum thickness, InP(001) slabs were generated for the subsequent surface studies. These slabs comprised 6 In and 6 P layers, where the bottom 5 bilayers were fixed to ideal bulk positions, and a vacuum of approximately ≈36 Å added along the surface normal. All surface calculations were conducted using an asymmetric slab, with the bottom In-layer being passivated with pseudo-hydrogen atoms (*Z* = 1.25). For *k*-space integration, 4 × 4 × 1 and 4 × 2 × 1 *k*-grids were selected, depending on the respective surface size.

### 2.2 Surface phase diagrams

To access the stability of different surface reconstructions with respect to each other, the Gibbs free surface energy, *γ* = Δ*G*/*A*, was determined following eqn (1).1
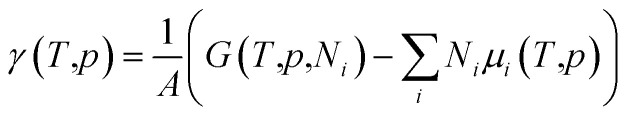
Here, *T* and *p* represent temperature and pressure, whereas *N*_*i*_ and *μ*_*i*_ correspond to number and chemical potential of species *i*, respectively*.* While *G* in principle depends on temperature and pressure, it is often simply approximated in ab initio-based approaches by the calculated total energy of the corresponding slab, thus neglecting entropy and volume change. Moreover, for the sake of representation, the chemical potential is frequently normalised with respect to the bulk energies of the respective elements (Δ*μ*_*i*_(*T*, *p*) = *μ*_*i*_(*T*, *p*) − *E*_*i*_), thus yielding:2
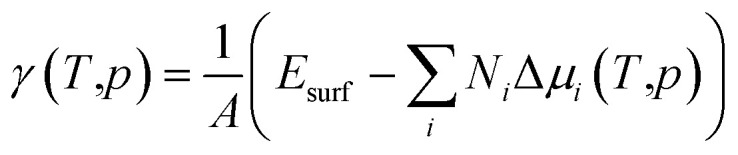


Next, the fact that, in equilibrium, *μ*_In_ and *μ*_P_ are connected *via* the bulk formation energy of InP, can be exploited:

3 *μ*_In_ + *μ*_P_ = *μ*_InP,bulk_

4 = *μ*_In,bulk_ + *μ*_P,bulk_ − Δ*H*^InP^_f_

Now, the surface free energy of the plain surfaces can be expressed solely as a function of Δ*μ*_In_.^25^

The above expressions (see eqn (4)) can furthermore be used to determine the limits for Δ*μ*_In_ (and Δ*μ*_P_):

5 Δ*H*^InP^_f_ ≤ Δ*μ*_In_ ≤ 0

The experimentally determined value for the InP formation energy corresponds to Δ*H*^InP^_f_ = −0.81 eV.

Starting from the clean surface reconstructions, the phase stability upon oxygen adsorption can consequently be represented as a function of Δ*μ*_In_ and Δ*μ*_O_. Here, it should be noted that the oxygen chemical potential can be expressed as a function of partial pressure, *p*, and temperature, *T*, as represented by the following equation:6
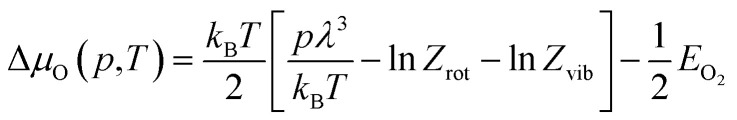
where *k*_B_ is the Boltzmann constant, *λ* is the de Broglie thermal wavelength of the O_2_ molecule, and *Z*_rot_, *Z*_vib_ are the rotational and vibrational partition functions of O_2_, respectively. *E*_O_2__ represents the energy of the oxygen molecule in its computational spin-triplet ground state.

## 3 Results & discussion

### 3.1 Phase stability – plain surface

**Fig. 1:**
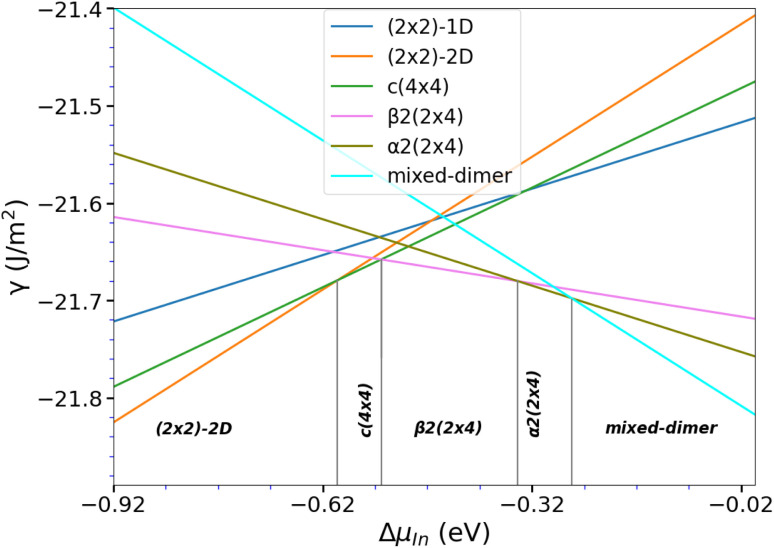
Surface free energy for the different InP(001) reconstructions as function of the indium chemical potential.

**Fig. 2:**
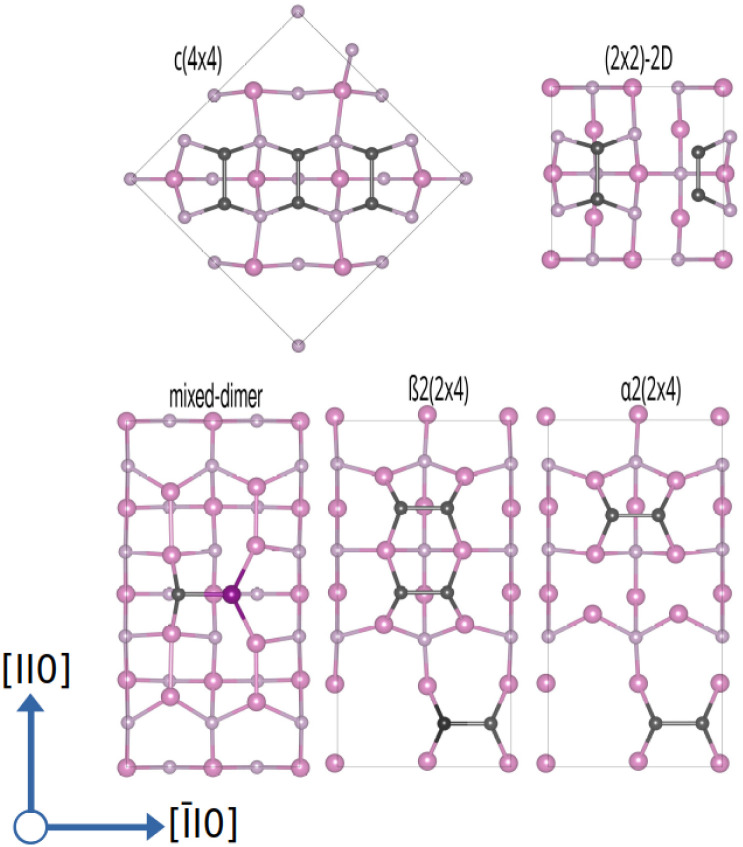
Ball-and-stick representation for stable InP(001) surface reconstructions, viewed along the (001) surface normal. Surface dimers, *i.e.* P-dimers are indicated in dark grey, and In–P dimers are indicated in a magenta-dark grey format. In and P atoms are indicated by pink and grey respectively.

As a first step, surface reconstructions of P-rich and In-rich phases, known to occur in InP and/or related compounds,^30–32^ were re-examined to determine the surface phase diagram of InP(001) with the inclusion of dispersion corrections. Plausible structures that have been found to be stable in earlier studies were extracted from the literature and subsequently optimised. The ground state energies of the optimised structures were then used to determine the surface phase diagram as a function of the indium chemical potential as shown in Fig. 1. Five stable surface reconstructions are observed (see Fig. 2). The (2 × 2)-2D reconstruction is stable for a large range under P-rich conditions. For increasing Δ*μ*_In_, the closely related *c*(4 × 4) phase becomes stable in a narrow potential range. Intermediate In chemical potentials stabilise the stepped β2(2 × 4) and α2(2 × 4) phases, whereas the mixed-dimer surface is observed for In-rich conditions. On the contrary, the (2 × 2)-1D surface is found to be slightly unstable in contrast to previous reports in the literature.^32^ This is most likely a consequence of the different exchange-correlation functional (LDA in their work, GGA in ours) as well as the fact that our work accounts for dispersion correction *via* the vdW-D3 scheme. For comparison, the phase-diagram was also recalculated, using LDA, to recognise the differences due to different classes of XC functional as previously reported.^32^ The instability of (2 × 2)-1D reconstruction for PBE could be due to the inaccurate correlation energy calculation using LDA for inhomogeneous systems. In general, the results obtained *via* the here applied PBE-D3 approach are expected to be more reliable. However, it has to be noted that the observed differences to the previously published LDA phase diagram are small and that additional uncertainties, as *e.g.* entropic effects, are not considered.

Finally, the unstable – no hydrogen termination was taken into account – P-rich (2 × 2) surface is also shown, as this surface becomes important as basis structure for the oxygenation study presented below.

The above-discussed surface reconstructions are depicted in Fig. 2. As these will be the starting point for the calculations in the presence of oxygen, their structural features will be quickly discussed. The P-rich (2 × 2)-2D and *c*(4 × 4) surfaces are terminated by additional P-dimers that are located on the already P-terminated InP(001) surface. In the case of the (2 × 2)-2D surface, two dimers that differ in length and position with respect to the underlying P-layer are observed. The closely related *c*(4 × 4) reconstruction is based on a 
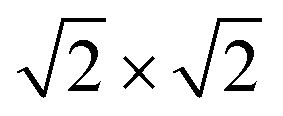
 surface unit cell that is rotated by 45° wrt. the conventional one. This surface reconstruction corresponds to a P-terminated surface as well, however, with an additional row of three P-dimers on top. The β2(2 × 4) phase can be described as a stepped surface with In-termination, with two P-dimers on the upper and one on the lower plateau. The α2(2 × 4) surface reconstruction, on the other hand, is based on the same stepped surface, however, with one P-dimer on top of and one below the step. Similarly, the P-rich (2 × 2) structure corresponds to a flat, In-terminated surface with P-dimers on top. This structure forms when a P-terminated surface is optimised and should not be confused with the above-discussed (2 × 2)-2D phase, where additional P-dimers are present. Finally, the mixed-dimer reconstruction corresponds to an In-rich surface, again based on a (2 × 4) surface unit cell with a single mixed-dimer on top of the In-termination.

### 3.2 Phase stability – oxygenated surface

**Fig. 3:**
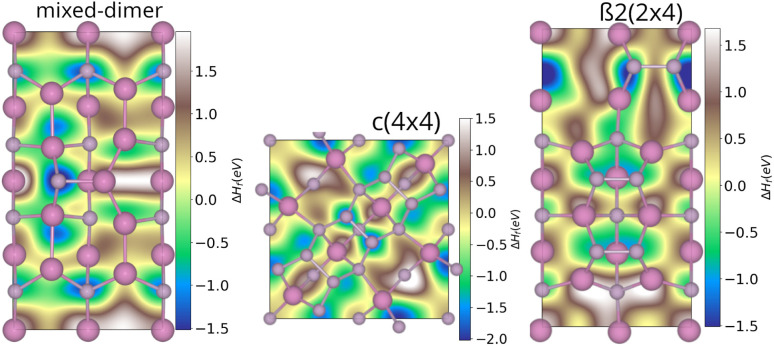
Potential energy surfaces for oxygen adsorption on different InP(001) surface reconstructions. In and P atoms were constrained, whereas O was allowed to relax along the surface normal.

Starting from the clean phase diagram, the above-mentioned stable reconstructions were investigated in the presence of oxygen. As a first step, the potential energy landscape of the respective surfaces was scanned by placing single oxygen atoms on a grid of equidistant points. The oxygen atoms were then relaxed along the surface normal, while the surface was kept fixed, thus yielding first insights with respect to the most likely adsorption sites. For the In-rich mixed-dimer surface, the energetically most favourable adsorption site was found to be the P-atom of the InP-dimer (see Fig. 3). This is in contrast with earlier findings on AlInP, where In was found to be more favourable for the mixed-dimer surface.^16^ In addition, the In atoms next to the P atom of the InP dimer are likely to form In–O–In bonds, also showing negative adsorption energies. Finally, the P atoms below the top-most In layer, that are directly accessible, offer favourable adsorption sites indicating the formation of In–O–P bonds with neighbouring In atoms. For all P-rich surfaces, adsorption minima were found to be preferentially on P-dimer sites. This is exemplified for the β2(2 × 4) and *c*(4 × 4) phases in Fig. 3. Interestingly, for β2(2 × 4), the most favourable adsorption sites are on the P-dimers of the lower plateau, which can be explained by the vicinity of the step edge. The dimers on the upper plateau are also favourable adsorption sites, however, to a smaller extent. For the *c*(4 × 4) surface, the P-dimer sites, but also the P-atoms of the P-layer below, are favourable adsorption sites. Thus, it can be concluded that the P-sites are particularly reactive towards oxygen, even for In-rich surfaces.

**Fig. 4:**
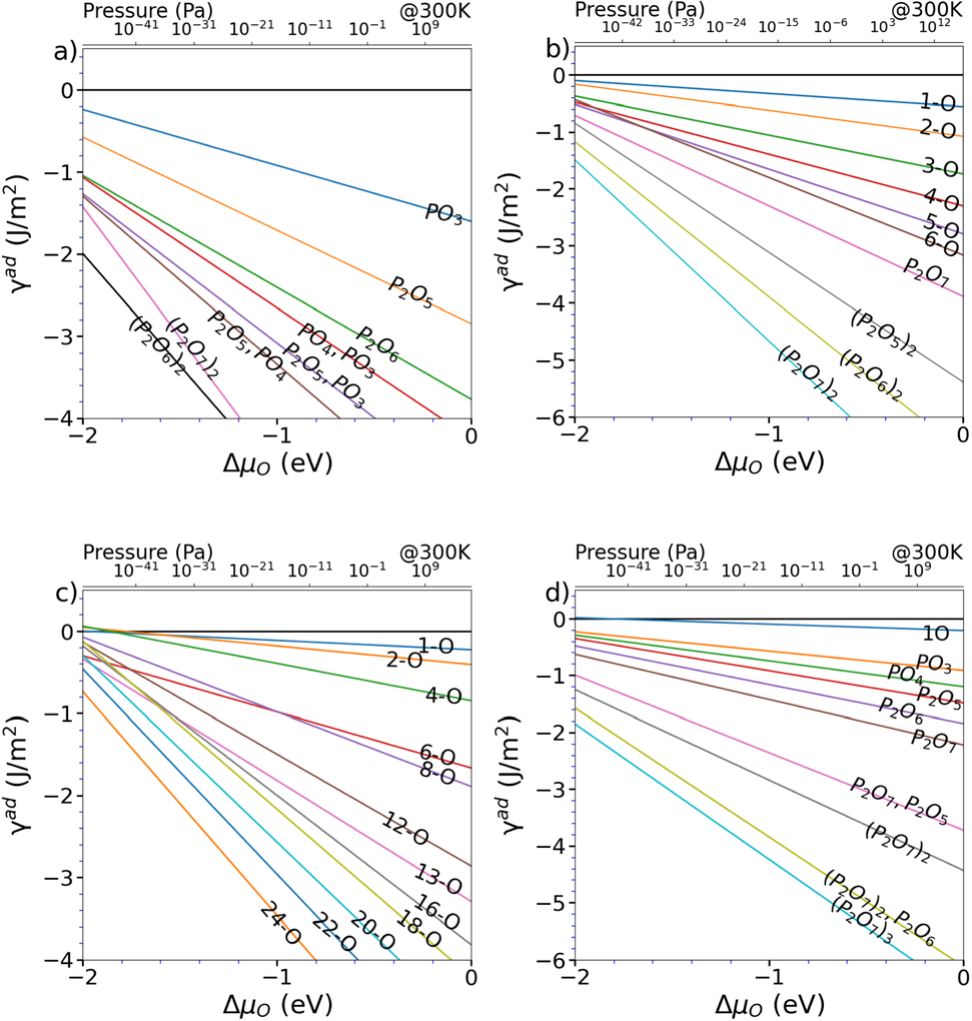
Surface energy wrt. oxygen chemical potential of the energetically favourable O adsorption sites for (a) (2 × 2), (b) (2 × 2)-2D, (c) mixed-dimer and (d) β2(2 × 4) surface reconstructions.

**Fig. 5:**
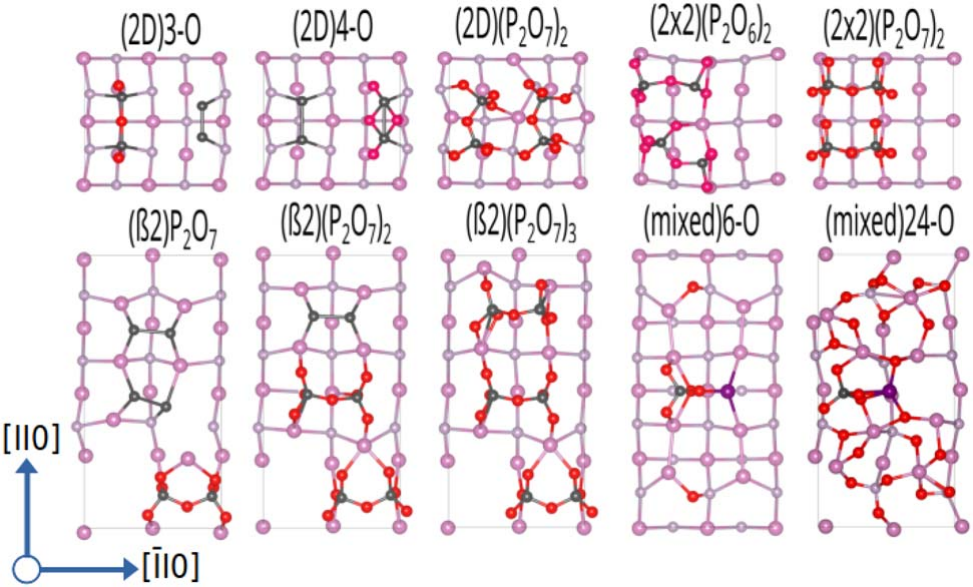
Ball-and-stick representation for InP(001) oxygenated surface reconstructions, viewed along the (001) direction. To distinguish between the types of dimers, surface P-dimers are shown in dark grey, and In–P dimers are represented with a magenta-grey pattern. In, P, and O atoms are shown in pink, grey, and red, respectively.

Starting from the such determined minima, the surface was also allowed to relax and the amount of surface oxygen was then gradually increased. For the In-rich, mixed dimer surface, in accordance to literature, a screening-based increase of the oxygen content resulted in a rather uniform oxygen distribution. This is due to the fact that In–O–P and In–O–In bonds are formed preferentially, as already indicated by the colour map in Fig. 3. While the structures with rather low oxygen coverage keep the symmetry of the underlying mixed dimer surface, this symmetry is getting lost at higher oxygen concentration. Increasing the coverage beyond 12 oxygen atoms results in more strongly disordered arrangements on the surface as can be inferred from the (mixed) 24-O-structure in Fig. 5 and the supplementary dataset.^33^ To evaluate the stability of the structures with different oxygen coverage, the corresponding phase diagram – depicting the surface free energy with respect to the oxygen chemical potential – were determined (see Fig. 4c). This clearly shows that oxygen-rich surfaces are rapidly stabilised with increasing Δ*μ*_O_, with the surface that features the highest considered oxygen loading being stable over a wide range. Only at very low oxygen chemical potentials (Δ*μ*_O_ < −2 eV), some of the ordered low coverage phases become stabilised. However, at room temperature, such low chemical potentials would correspond to extremely low oxygen partial pressure, thus making their occurrence rather unlikely.

Interestingly, the oxygenation of the In-terminated surfaces with P-dimers on top was found to follow a different path. For example, for the case of the β2(2 × 4) phase, surface configurations with increased oxygen content were obtained by sequentially determining the most favourable position for each added oxygen. This resulted in the preferential formation of P–O and In–O–P bonds on P-dimers. However, at increased oxygen content, the occurrence of characteristic P_*x*_O_*y*_ polyphosphate-type moieties was observed. The observation of these polyphosphates resulted in the question if such motifs might be stable also at lower oxygen contents. Consequently, an additional approach, based on the decoration of the surface with such structural motifs was followed. For this purpose, isolated PO_3_, PO_4_, P_2_O_5_, P_2_O_6_, and P_2_O_7_ motifs and combinations thereof were investigated and indeed proved to be more stable than the previously obtained structures with a more uniform distribution of oxygen. While the different polyphophate moieties are in general observed to be the most stable structures for a given oxygen content, the P_2_O_7_-based motifs are found to be particularly stable configurations (see Fig. 5). A subset of these structures was also suggested by a study of Zhang *et al.*,^21^ based on the interpretation of photoelectron spectroscopy data. The reason for this motif formation lies in the fact that oxygen strongly prefers to form bonds with phosphorous, such that P–O, P–O–P and predominantly P–O–In bonds are formed at higher oxygen content. Consequently, the number of available P-dimers on the β2(2 × 4) surface limits the polyphosphate formation, thus reaching its maximum when all three P-dimers are part of a P_2_O_7_ motif, corresponding to a surface with 21 adsorbed oxygen atoms, in the following also referred to as 21-O surface. With respect to the surface free energy, as in the case of the mixed dimer, the oxygen rich surfaces are, already at low values for Δ*μ*_O_, dominating the phase diagram, with the maximum coverage of three P_2_O_7_ units being the most stable configuration over a wide chemical potential range (see Fig. 4d).

The α2(2 × 4) phase behaves in a similar fashion, the limiting concentration for polyphosphate formation here does, however, correspond to the 14-O surface, which shows P_2_O_7_ motifs on both available P-dimers. Increasing the oxygen content of the α2(2 × 4) phase beyond 14-O leads to an equivalent distribution of O-atoms around In sites. This results in the formation of In–O–In bonds with a disordered underlying In-layer.

As β2(2 × 4) and α2(2 × 4) correspond to stepped surfaces that are terminated by P-dimers, the question on the potential stability of a fully P-terminated, flat surface arises. For this purpose, the P-rich (2 × 2) surface, which previously was found to be unstable, was investigated for oxygen adsorption. Interestingly, P_2_O_6_ and P_2_O_7_ motifs forming additional In–O–In bonds in the In-termination are observed as most stable entities. The saturation of both P-dimers with oxygen results in the (2 × 2) phase, containing 14 oxygen atoms (14-O surface) as limiting case, as shown in Fig. 5. It has to be noted that this corresponds to a 28-O coverage for a (2 × 4) surface. With respect to the surface free energy, we again see a stabilisation of the structures with increased oxygen content already at low oxygen chemical potential. The maximum coverage of two P_2_O_7_ motifs is the energetically most favourable structure over a wide chemical potential range, down to Δ*μ*_O_ ≈ −1.75 eV.

When considering the P-rich phases, the (2 × 2)-2D surface exhibits a strong preference for the formation of pholyphosphate moities (see Fig. 5). This involves the insertion of oxygen into the underlying In-layer of a P-terminated 2-P-dimer surface forming an In–O–P bond. As for the previous cases, P_2_O_7_ motifs were found to be the limiting case. This corresponds to a maximum of 14 oxygen (14-O phase) atoms when both top P-dimers are part of a P_2_O_7_ moiety. When the surface energy is considered, the oxygen-rich phases are again dominant, with the 14-O surface, *i.e*. (2 × 2)-2D(P_2_O_7_)_2_, being the most stable one down to Δ*μ*_O_ ≪ −2.5 eV (see Fig. 4b).

### 3.3 Phase stability – overall phase diagram

**Fig. 6:**
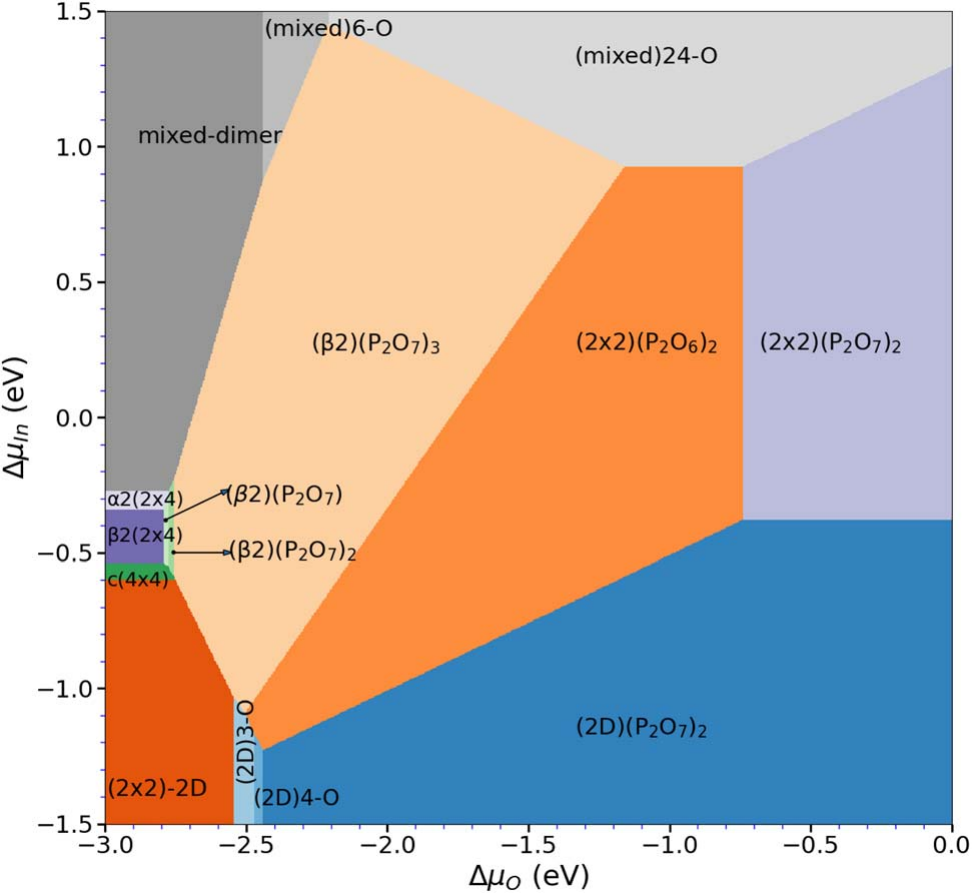
Overall phase diagram of the oxygenated InP(001) surface, showing the most stable surface reconstructions as function of the chemical potentials of indium and oxygen.

After assessing the phase diagrams of the different surface reconstructions with respect to the oxygen chemical potential, the question on the respective stability of these phases remains. For this purpose, we have determined the overall phase diagram, taking the chemical potentials of indium and oxygen into account (see eqn (2)). Indeed, already at rather low oxygen chemical potential, oxygenated surfaces become quickly more stable than the pristine clean surface reconstructions. However, the stability range of the different phases shifts significantly on the Δ*μ*_In_ scale as can be seen in Fig. 6. In fact, starting from the clean surfaces, the β phase becomes dominant at rather low oxygen chemical potential covering a wide range of Δ*μ*_In_ values. For increasing Δ*μ*_O_, meaning more oxygen-rich conditions, (2 × 2)-based phases become more stable. This is simply due to the fact that these surfaces can adsorb one additional P_2_O_7_ motif per (2 × 4) surface unit before reaching saturation. At low Δ*μ*_In_ (≤ −1.09 eV) and for Δ*μ*_O_ ≤ −2.45 eV, P-rich, (2 × 2)-2D-based structures with low oxygen content become stable. In general, lower Δ*μ*_O_ shifts P-rich surfaces to lower Δ*μ*_In_ values. Conversely, for larger Δ*μ*_O_, the P-rich, (2 × 2)-2D based surface expands its region of thermodynamic stability. Furthermore, apart from the clean surface, mixed dimer-based structures are only stabilised for largely positive Δ*μ*_In_ (≥0.85 eV) values. This is already far in the range, where In-metal should form and therefore it seems unlikely that mixed-dimer- or α2(2 × 4)-based structures are observable in the presence of oxygen. This is a rather surprising finding, as experimental and computational studies on InP-based surfaces have usually focused on the mixed dimer structure and derivatives thereof.^16^ In fact, the here presented results clearly show that the formation of PO_*x*_-based motifs is the dominant mechanism on the InP(001) surface, thus determining the surface passivation in the presence of oxygen.

## 4 Conclusions

In conclusion, this paper re-investigated the stability of the (2 × 2)-2D- and *c*(4 × 4)-based surface reconstructions in the P-rich domain *via* DFT calculations with the inclusion of van der Waals interactions. The surface reconstructions β2(2 × 4) and α2(2 × 4) are observed for intermediate chemical potential, while for the In-rich domain, the mixed-dimer reconstruction is observed.

The introduction of oxygen changes the picture significantly. While P-rich phases strongly favour the formation of (P_*x*_O_*y*_) polyphosphate moieties, with P_2_O_7_ motifs being particularly stable, the In-rich mixed dimer phase shows a rather homogeneous distribution of oxygen on the surface. In the overall phase diagram, this results in the finding that the typically investigated mixed-dimer reconstruction and ordered derivatives thereof are unlikely to be observed in the presence of oxygen. This corroborates experimental findings,^15^ where oxygen adsorption on InP in vacuum was found to turn the surface optically isotropic starting from the mixed-dimer reconstruction, but not for the P-rich, (2 × 2)-2D–2H surface. This is an important finding as the mixed-dimer surface has typically been considered as being the dominant surface reconstruction in the presence of oxygen, probably because this surface reconstruction is experimentally more easily accessible *via* sputtering–annealing routines. Our results now show that P-rich phases are stable over a wide range of Δ*μ*_In_ (and Δ*μ*_P_), which means that for instance in electrochemical environments, these phases can be expected to be observed, whereas ordered mixed-dimer based phases seem very unlikely.

## Data availability

The data that support the findings of this study, including structure files of the most stable structures, are openly available on NOMAD at https://doi.org/10.17172/NOMAD/2025.03.11-2.^33^

## Conflicts of interest

There are no conflicts to declare.
